# Evaluation of the hepatitis C cascade of care among people living with HIV in New South Wales, Australia: A data linkage study

**DOI:** 10.1111/jvh.13658

**Published:** 2022-02-25

**Authors:** Samira Hosseini‐Hooshyar, Maryam Alavi, Marianne Martinello, Heather Valerio, Shane Tillakeratne, Gail V. Matthews, Gregory J. Dore

**Affiliations:** ^1^ The Kirby Institute UNSW Sydney Sydney New South Wales Australia

**Keywords:** care cascade, direct‐acting antivirals, HCV, HCV RNA testing, people living with HIV

## Abstract

People living with HIV (PLHIV) are a priority population to receive hepatitis C virus (HCV) screening and treatment. We aimed to characterize the HCV care cascade among PLHIV between 2010 and 2018 and to compare HCV testing and treatment uptake pre‐ and post‐availability of direct‐acting antivirals (DAAs) in New South Wales (NSW), Australia. Records of all HCV notifications (1993–2017) were linked to HIV notifications, deaths, hospitalizations, incarcerations, opioid agonist therapy, HCV RNA testing and treatment databases. Numbers and proportions were calculated for all stages of the care cascade and factors associated with HCV testing, and DAA treatment uptake were evaluated using logistic regression. From 383 individuals with HCV notification (2009–2017), 349 (91%) were ever HCV RNA tested, 285 (74%) had an indicator of chronic HCV infection, and from those eligible for treatment, 210 (74%) received HCV treatment. HCV testing was recorded for 85% pre‐DAA era and reached a cumulative proportion of 90% post‐DAA while treatment uptake had a 10‐fold increase from 7% pre‐ to 73% post‐DAA era. Younger age (adjusted odds ratio [aOR] 0.98; 95% CI 0.96–0.99), female gender (aOR 1.87; 95% CI 1.10–3.19), and rural region residence at notification (aOR 1.56; 95% CI 1.03–2.36) were associated with not receiving HCV testing. No identified factor was associated with not receiving treatment post‐DAA era. Removing barriers to HCV testing, expanding treatment to a variety of settings and continuous education and harm reduction are essential to achieve HCV elimination among PLHIV in Australia.

## 1 INTRODUCTION

An estimated 2.3 million people worldwide are living with HIV/hepatitis C virus (HCV) coinfection.^1^ Consequences of HCV infection are more severe among people living with HIV (PLHIV) including increased risk of cirrhosis, hepatic failure and hepatocellular carcinoma.^2^, ^3^ Further, liver‐related mortality has become one of the most frequent causes of death among PLHIV who have access to combination antiretroviral therapy.^4^ PLHIV have therefore been identified as a priority population to receive HCV screening and treatment,^5^ in efforts to meet the World Health Organization (WHO) HCV elimination targets. These targets include diagnosing 90% of people living with HCV infection and treating 80% of those diagnosed by 2030.^6^ Accordingly, for Australia to achieve the WHO HCV elimination goals, improvements in diagnosis and treatment of HCV PLHIV are essential.

Treatment for HCV has undergone tremendous changes from a period of moderately effective interferon‐based treatments to highly effective direct‐acting antiviral (DAA) therapies. In the DAA era, people living with HIV/HCV coinfection tend to achieve high sustained virological response (SVR; ≥95%) rates, comparable to those obtained among HCV mono‐infected individuals.^7^, ^8^ In Australia, government‐subsidized DAA therapy has been available since March 2016 for all adults with HCV infection, including PLHIV.^9^, ^10^ Unrestricted access to DAA therapy, at no or minimal cost to the individual, has facilitated rapid HCV treatment scale‐up among PLHIV.^11^, ^12^ However, for treatment scale‐up to continue having a great impact, screening efforts must reach undiagnosed individuals, and new diagnoses must be linked with care and treatment.^13^

To assess progress towards WHO HCV elimination targets, monitoring HCV‐infected individuals across the care continuum or cascade of care is imperative.^14^ An evaluation of the current HCV care cascade can provide a useful benchmark for tracking the effectiveness of programs and interventions and identify the service and access gaps at a broad population level.^15^ The objectives of this study were to characterize the HCV care cascade among PLHIV in New South Wales (NSW), Australia, between 2010 and 2018, and to compare HCV RNA testing and HCV treatment uptake among PLHIV pre‐ (2010–2015) and post‐introduction (2016–2018) of DAAs in Australia. A further objective was to evaluate factors associated with not receiving HCV RNA testing and DAA treatment uptake among PLHIV in NSW, Australia.

## 2 METHODS

### 2.1 Study setting

Australia is one of the few settings globally with well‐established infrastructure for linking positive HCV serology notifications to administrative databases.^16^ This study was conducted in NSW; Australia's most populous state with over 7 million inhabitants. In 2017, around 32% of HIV notifications (310/963) and 39% of hepatitis C notifications (4078/10,537) in Australia occurred in NSW.^17^ Further, an estimated 2290 individuals (uncertainty range: 1920–2690) were living with HIV/HCV coinfection in Australia in 2016.^18^

### 2.2 Data sources and record linkages

HIV and HCV are both notifiable diseases in NSW, Australia. Records of all individuals with HCV positive serology are held with the NSW Notifiable Conditions Information Management System (NCIMS) since 1993. Further, records of all new HIV diagnoses are maintained with National HIV Registry since 1985. A notification of both positive HCV and HIV serology (i.e. people living with HIV/HCV coinfection) was the study inclusion criteria.

Data linkage occurred in two stages. First, records of all individuals with HCV positive serology from NCIMS were linked to the (1) National HIV Registry, holding data from 1985, (2) Perinatal Data Collection (PDC Mothers) dataset, holding data from 1994, (3) NSW Admitted Patient Data Collection (APDC) database, holding data from 2001, (4) NSW Registry of Births, Deaths, and Marriages (BRDM), for date of death from 1993, (5) NSW Electronic Recording and Reporting of Controlled Drugs system (ERRCD), holding data from 1985 and (6) NSW Bureau of Crime Statistics and Reporting Corrective Services Custody Database (BOCSAR Custody) dataset, holding data from 1994. The NSW Centre for Health Record Linkage (CHeReL) used demographic details (including full name, sex, date of birth and address) to link records probabilistically and deterministically between the above‐mentioned datasets. However, linkage between NCIMS and the National HIV Registry dataset occurred using name codes (first two letters of last and first names) of those notified for enhanced privacy protection. CHeReL then provided the above‐described set of identifiers for the NCIMS cohort to the Integration Services Centre at the Australian Institute of Health and Welfare (AIHW). AIHW conducted the second round of linkage between NCIMS records and Medicare Benefits Schedule (MBS) dataset holding dates of HCV RNA testing from 2010 and Pharmaceutical Benefits Schedule (PBS) dataset holding HCV therapy dispensing data from 2010.

### 2.3 Study period

For the study period, data extractions occurred from each database as follows: HCV notifications (1 January 1993–31 December 2017); HIV diagnoses (1 January 1985–31 December 2017); hospitalizations (1 July 2001–30 June 2018); deaths (1 January 1993–30 June 2018); opioid agonist therapy (OAT) dosing (1 January 1985–19 September 2018); incarcerations (1 January 1994–31 December 2017); and HCV RNA testing and treatment (1 April 2010–31 December 2018).

### 2.4 Study outcomes

The primary outcomes were HCV RNA testing and HCV treatment uptake. People who have received an RNA test included those with RNA testing records in MBS dataset (first time counted), people with genotype testing records in MBS dataset (first time counted), and people who have been prescribed and dispensed HCV treatment in PBS dataset. Subsequent records of diagnostic testing or genotype testing were not included in the analyses.

HCV treatment uptake was estimated among those with an indicator of chronic HCV infection. Individuals with an indicator of chronic HCV infection were defined as those with records of genotype testing in MBS dataset or people who were dispensed HCV treatment in PBS dataset. HCV treatment uptake was defined by the date of first HCV treatment dispensing (interferon or DAA therapy) in the PBS dataset. Subsequent dispensing records were not included in the treatment uptake analyses, unless retreatment occurred in the DAA era.

### 2.5 Study population and inclusion criteria

For analysis of HCV RNA testing and treatment uptake in the pre‐DAA era (2010–2012 and 2013–2015) and post‐DAA era (2016–2018), all individuals who were alive for at least the first six months of each follow‐up period were included. Individuals also needed to be at least 18 years old by the end of each follow‐up period (i.e. 18 years old by 31 December 2012, 31 December 2015 and 31 December 2018).

### 2.6 Exclusion criteria

As the target population of this study were people living with HIV/HCV coinfection, records of HCV mono‐infection were excluded. Duplicate records of HCV notifications were also excluded. To allow time for treatment uptake, records were removed if death occurred before or in 2009. Records with no Medicare number were excluded. For analyses of HCV RNA testing and treatment uptake, only HCV notifications occurring during or after 2009 were included.

### 2.7 Exposure variables

Year of birth, gender (male, female), and local health district (LHD) of residence at time of HCV notification (metropolitan [metro], outer‐metro, and rural),^19^ were obtained from NCIMS. Identification of Aboriginal and Torres Strait Islander Peoples (hereafter referred to as Aboriginal) was obtained using PDC Mothers dataset. However, since the reporting of Aboriginal identification in administrative data was suboptimal, an algorithm developed by NSW Health^20^ was applied across datasets to identify Aboriginal Peoples. History of hospitalizations occurring due to alcohol‐use disorder^21^ and injecting drug use (IDU) was obtained using APDC database. Alcohol‐use disorder is a standard term used to describe continued drinking despite adverse mental and physical consequences.^21^ All hospitalization records were coded using the 10th revision of the Classification of Diseases and Related Health Problems (ICD‐10). As previously described,^22^ a hospital discharge diagnosis code (ICD‐10) was used to infer the presence of alcohol‐use disorder (Table S1). IDU‐related hospitalizations (i.e. hospitalizations occurring due to injectable drugs and/or infections indicative of injection drug use) were also identified using the ICD‐10 classifications of disease manual (Table S2). Drug dependence was defined by combining IDU‐related hospitalizations from APDC database and/or receipt of OAT dosing from ERRCD, as previously described.^23^ IDU‐related hospitalizations and/or receipt of OAT occurring between 2016 and 2018 were considered as indicators of recent drug dependence; records with last hospitalization or OAT dosing recorded any time pre‐2016 were considered as indicators of distant drug dependence; and records with no hospitalization or OAT dosing were considered as indicators of no evidence of drug dependence. Recent incarceration defined as experiencing any length of imprisonment during the DAA era (2016–2018) was obtained from the BOCSAR Custody dataset.

### 2.8 Statistical analysis

Characteristics of people with an HCV/HIV coinfection notification were described overall and by outcomes: HCV RNA testing and HCV treatment uptake.

Number and proportion of PLHIV for the following steps of HCV care cascade between 2010 and 2018 were calculated: (a) HCV notification; (b) HCV RNA testing; (c) chronic HCV infection (indication); and (d) HCV treatment uptake. Proportion of people with HCV RNA testing, chronic HCV infection (indication) and HCV treatment uptake were also calculated from the people in each preceding step. Number and proportion of people with HCV RNA testing and treatment uptake were also calculated and compared between pre‐ (2010–2015) and post‐DAA (2016–2018) era. Median time from HCV RNA testing (earliest HCV RNA testing date on record) to first treatment uptake on record were also calculated by each calendar year.

Unadjusted and adjusted logistic regression models were applied to evaluate factors associated with not receiving HCV RNA testing overall (2010–2018), and factors associated with not receiving DAA treatment uptake (2016–2018). All exposures with a *p*‐value less than .2 in the unadjusted model, or those that were known to be associated with each outcome were considered for inclusion in the adjusted model. All analyses were performed in STATA v.14.0 [College Station, TX, USA].

## 3 RESULTS

### 3.1 Characteristics of people living with HIV/HCV coinfection, 1993–2017

Between 1993 and 2017, 988 individuals were identified as living with HIV/HCV coinfection in NSW, Australia (Table 1). Median year of birth was 1966 (IQR 1960–1973), most were male (92%, *n* = 912) and born in Australia (76%, *n* = 679). Nine percent (*n* = 82) identified as Aboriginal and/or Torres Strait Islander. A total of 17% (*n* = 171) had a history of alcohol‐use disorder. Individuals were mostly HCV notified in metropolitan area (68%, *n* = 657), followed by rural (16%, *n* = 156) and outer‐metropolitan (15%, *n* = 147) areas. Only 5% (*n* = 47) had a history of incarceration in DAA era. Further, in DAA era (2016–2018), 61% (*n* = 598) had no evidence of drug dependence, 20% (*n* = 200) had evidence of distant drug dependence and 19% (*n* = 190) had evidence of recent drug dependence.

**TABLE 1:** Characteristics of people living with HIV/HCV coinfection in NSW, 1993–2017, *n* = 988

Characteristics, *n* (%)	Total *n* = 988	%	Ever received RNA testing, *n* = 751	%	Ever received HCV treatment, *n* = 419	%
Year of birth, median (IQR)	1966 (1960–1973)	–	1967 (1961–1973)	–	1968 (1961–1974)	–
Male sex	912	92	705	94	397	95
Born in Australia^a^	679	76	545	77	308	77
Aboriginal ethnicity^b^	82	9	61	9	34	9
History of alcohol‐use disorder	171	17	128	17	73	17
LHD of residence at the time of HCV^c^						
Metro	657	68	516	71	285	71
Outer metro	147	15	105	14	58	14
Rural	156	16	110	15	61	15
DAA era variables						
Incarcerated in 2016–2018	47	5	32	4	26	6
Drug dependence in 2016–2018						
Recent dependence	190	19	162	22	99	24
Distant dependence	200	20	163	22	100	24
No evidence of dependence	598	61	426	57	220	53

Abbreviations: DAA, direct‐acting antiviral; HCV, hepatitis C virus; HIV, human immunodeficiency virus; IQR, interquartile range; LHD, local health district; RNA, ribonucleic acid.

^a^ Among people with available data. 91 had missing.

^b^ Among people with available data. 117 had missing.

^c^ Among people with available data. 28 had missing.

### 3.2 Overall HCV cascade of care among people living with HIV/HCV coinfection (2010–2018)

**FIGURE 1:**
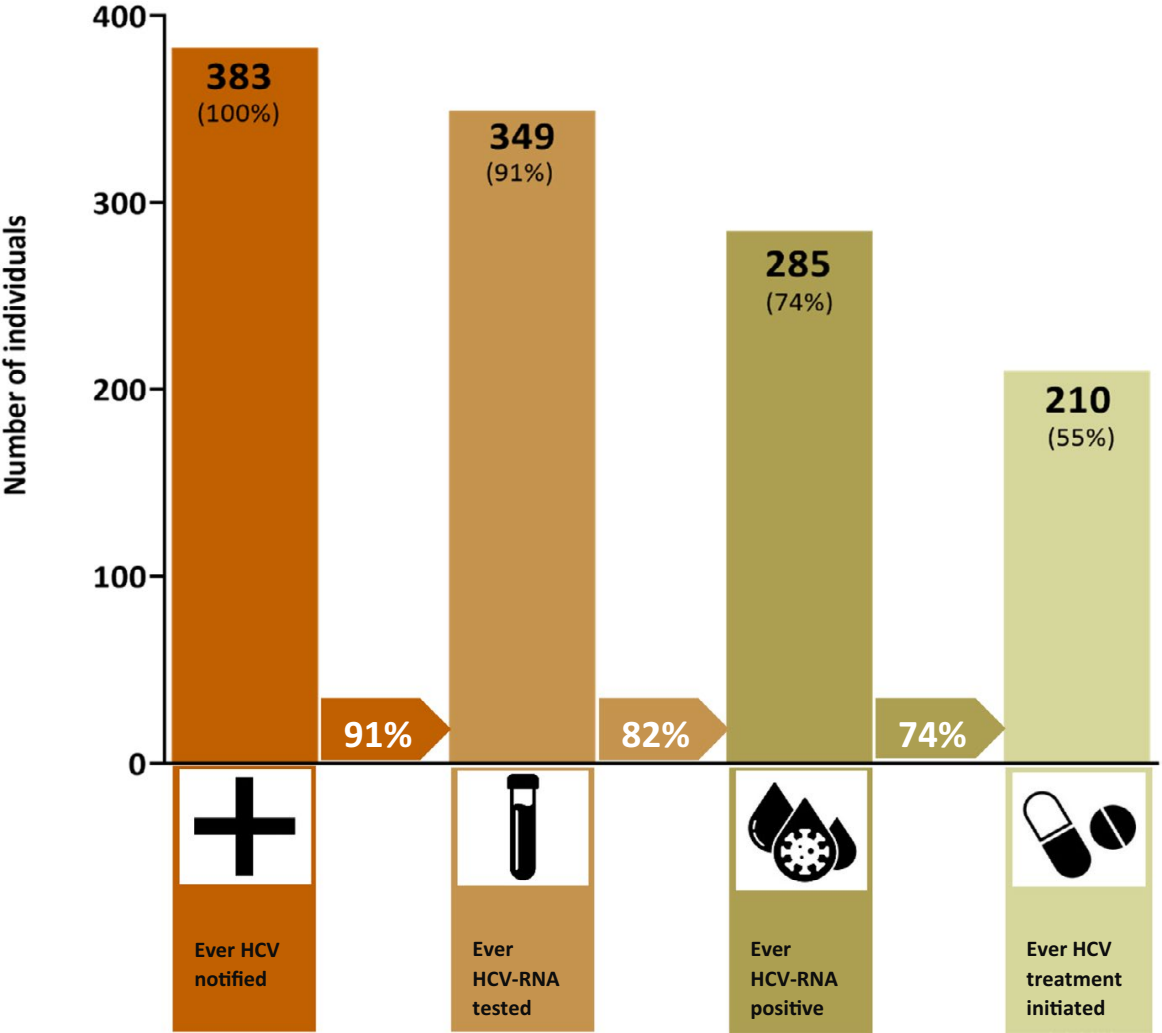
HCV cascade of care among people living with HIV/HCV coinfection, New South Wales, Australia, 2010–2018. Arrows between bars represent the proportion of patients in each step of the cascade from the patients in the preceding step. For example, 74% of those ever HCV‐RNA positive initiated HCV treatment

A total of 383 individuals were alive and HCV notified between 2009 and 2017 (Figure 1). Of this population, 349 (91%) ever received HCV RNA testing and 285 (74%) had an indicator of chronic HCV infection and were eligible for treatment. From the population eligible for treatment, 210 (74%) ever received HCV treatment.

### 3.3 HCV RNA testing and HCV treatment uptake—pre‐DAA era (2010–2015)

**FIGURE 2:**
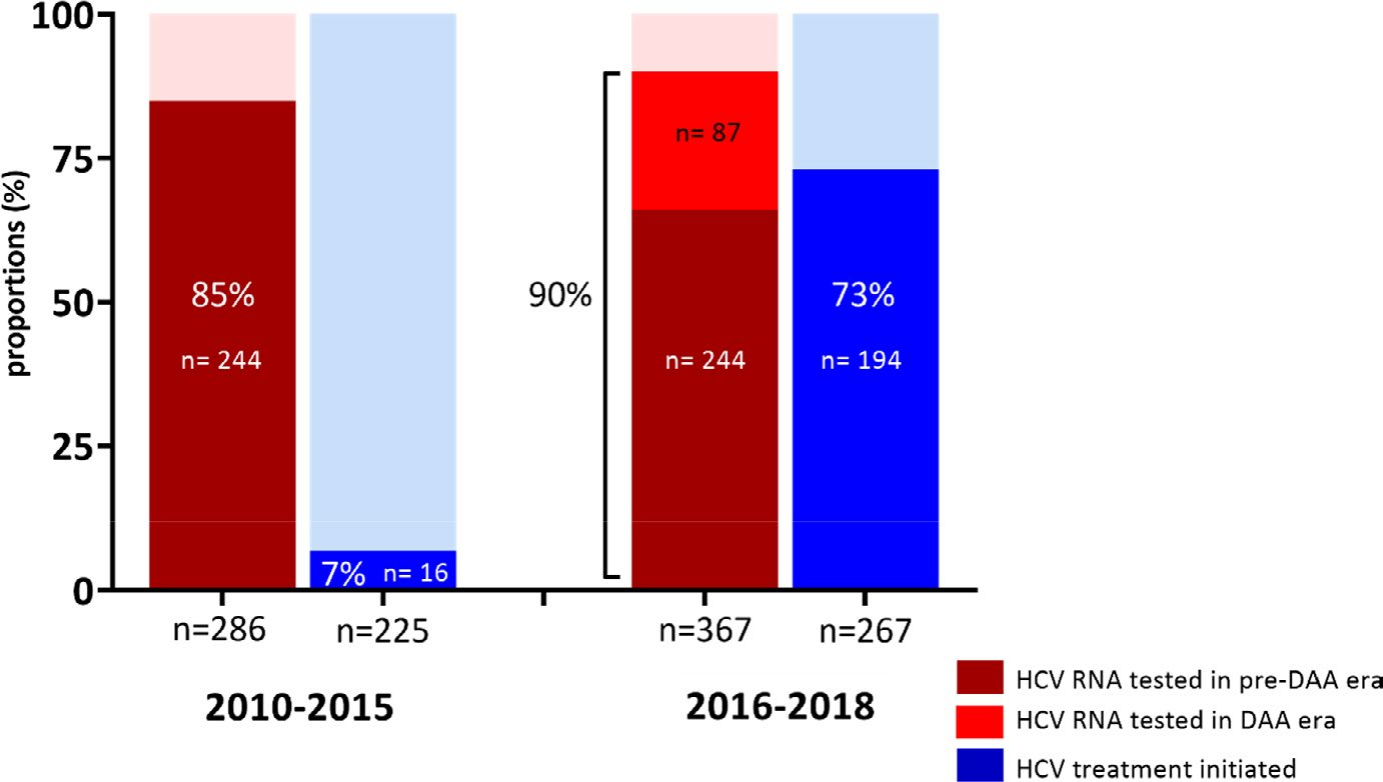
HCV RNA testing and treatment uptake in the pre‐DAA era (2010–2015) and post‐DAA era (2016–2018), among people living with HCV/HIV coinfection NSW, Australia

Between 2010 and 2015 (pre‐DAA era), a total 286 individuals were alive, and HCV notified, and therefore, could receive HCV RNA testing (Figure 2). Of this population, 244 (85%) received an HCV RNA test in pre‐DAA era. A total of 225 individuals had an indicator of chronic HCV infection and were eligible for treatment, of whom 16 (7%) received HCV treatment in pre‐DAA era.

### 3.4 HCV RNA testing and HCV treatment uptake—post‐DAA era (2016–2018)

To derive the DAA era population (2016–2018), people who received HCV treatment in pre‐DAA era (*n* = 16) with no subsequent treatment episodes were presumed to have cleared HCV through therapy and were excluded, resulting in 367 individuals; 286 notified in pre‐DAA era and 81 in DAA era. Of this population (*n* = 367), 244 received HCV RNA testing pre‐DAA era and 87 in DAA era resulting in 331 (90%) individuals ever receiving HCV RNA testing.

A total of 123 individuals were eligible for first HCV RNA testing in DAA era, including 81 individuals newly HCV notified in DAA era, and 42 individuals notified in pre‐DAA era who had never received RNA testing. Of this population (*n* = 123), 87 (71%) received HCV RNA testing. Among the 81 individuals newly notified in DAA era, 75 (93%) received HCV RNA testing.

A total of 267 individuals had indicators of chronic HCV infection in DAA era, of whom 194 (73%) received HCV treatment.

### 3.5 Time to HCV treatment uptake

**FIGURE 3:**
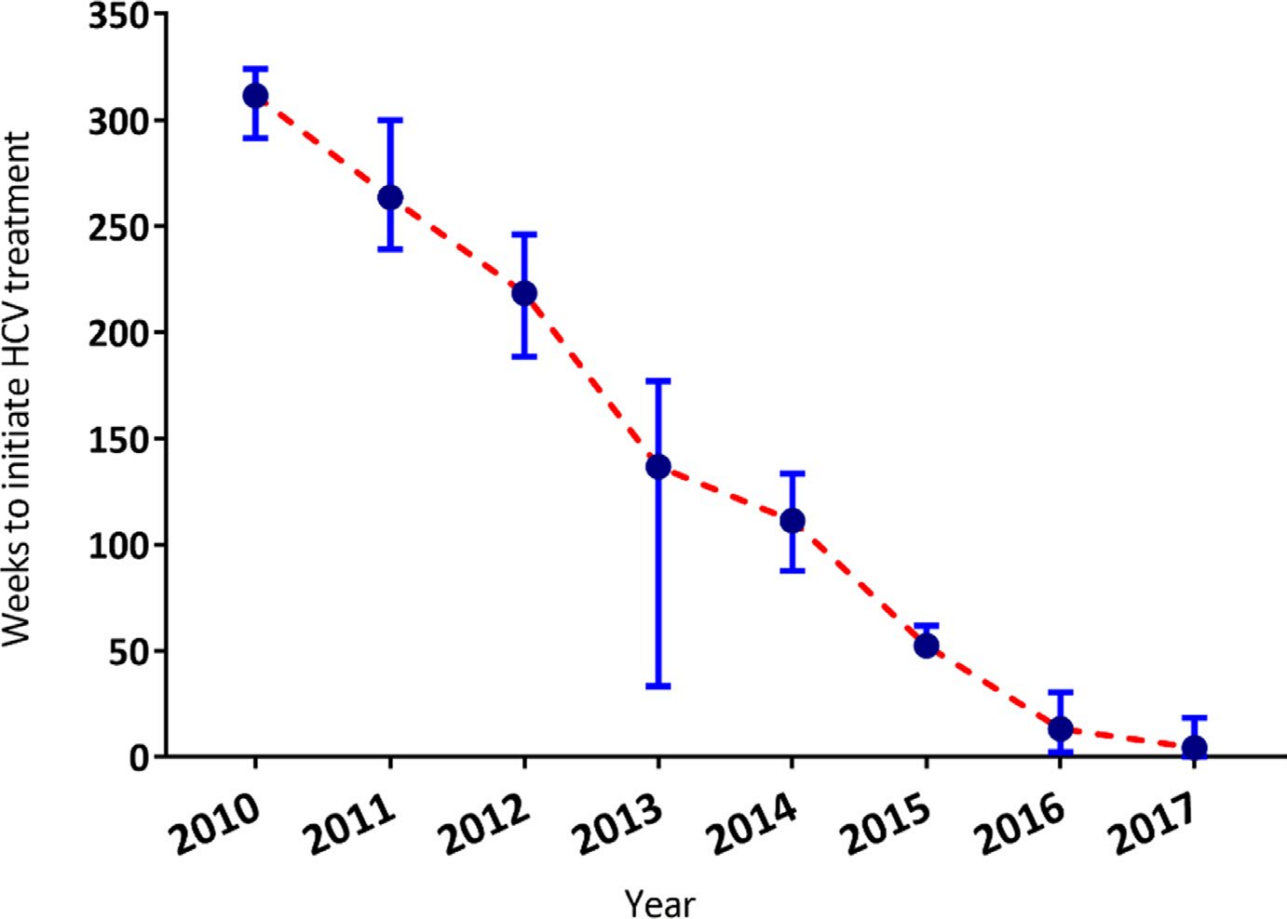
Median time from HCV testing to HCV treatment initiation among people living with HCV/HIV coinfection in NSW 2010–2017, *n* = 383

The median time from HCV RNA testing to HCV treatment uptake is shown in Figure 3, by year 2010 to 2017. Time to treatment uptake decreased from 311 weeks in 2010 to 52 weeks in 2015 (end of pre‐DAA era), further declining to four weeks (IQR 0–18) by 2017 (DAA era).

### 3.6 Factors associated with not receiving HCV RNA testing, ever (2010–2018)

After adjusting, not receiving HCV RNA testing was associated with younger age (adjusted odds ratio [aOR] 0.98; 95% CI 0.96–0.99, *p* = .023), female gender (aOR 1.87; 95% CI 1.10–3.19, *p* = .022), and rural region residence at HCV notification (aOR 1.56; 95% CI 1.03–2.36, *p* = .035) (Table 2).

**TABLE 2:** Unadjusted and adjusted analyses of factors associated with not receiving HCV RNA testing during 2010–2018, among people living with HIV/HCV coinfection in NSW, *n* = 988

Characteristics, *n* (%)	Not received HCV RNA testing		Unadjusted analysis		Adjusted analysis		
	*n* = 237	%	OR	95% CI	aOR	95% CI	*p*
Year of birth (median, IQR)	1965 (1959–1973)	_	0.98	(0.97–1.0)	0.98	(0.96–0.99)	.**023**
Sex							
Male	207	23	1.00		1.00		
Female	30	40	2.27	(1.39–3.70)	1.87	(1.10–3.19)	.**022**
Aboriginal ethnicity							
No	148	19	1.00		1.00		
Yes	21	26	1.49	(0.88–2.53)	1.50	(0.86–2.60)	.155
Country of birth							
Australia	134	20	1.00				
Overseas	51	23	1.24	(0.86–1.79)			
History of alcohol‐use disorder							
No	194	24	1.00				
Yes	43	25	1.08	(0.74–1.58)			
LHD of residence at the time of HCV							
Metro	141	21	1.00		1.00		
Outer metro	42	29	1.46	(0.98–2.19)	1.40	(0.91–2.16)	.124
Rural	46	29	1.53	(1.03–2.26)	1.56	(1.03–2.36)	.**035**
Incarcerated in 2010–2018							
No	182	23	1.00				
Yes	27	25	1.11	(0.7–1.78)			
Drug dependence in 2010–2018							
Yes	64	19	1.00		1.00		
No	173	27	1.59	(1.15–2.19)	1.25	(0.88–1.78)	.212

Abbreviations: HCV, hepatitis C virus; IQR, interquartile range; LHD, local health district; RNA, ribonucleic acid.

Bold indicates associations with *p*‐value less than .05 were considered statistically significant.

### 3.7 Factors associated with not receiving HCV treatment in the DAA era (2016–2018)

After adjusting, there were no factors associated with not receiving HCV treatment in the DAA era (Table 3). However, given the small number of untreated individuals in DAA era (*n* = 68), the sample size was underpowered.

**TABLE 3:** Unadjusted analysis of factors associated with not receiving HCV treatment in the DAA era (2016–2018), among people living with HIV/HCV coinfection in NSW, *n* = 262

Characteristics, *n* (%)	Not received DAA treatment, *N* = 68	%	OR	95% CI	*P*
Year of birth (median, IQR)	1969 (1963–1975)	_	0.99	0.96–1.02	.393
Sex	not included due to small *n* of female, *n *≤ 5				
Aboriginal ethnicity					
No	58	25	1.00		
Yes	7	41	2.06	0.75–5.67	.160
Country of birth					
Australia	48	27	1.00		
Overseas	18	26	0.98	0.52–1.85	.956
History of alcohol‐use disorder					
No	58	26	1.00		
Yes	10	27	1.06	0.49–2.34	.872
LHD of residence at the time of HCV					
Metro	54	27	1.00		
Non‐metro	12	21	0.72	0.35–1.47	.370
Incarcerated in 2016–2018	not included due to small *n* of people in custody, *n *≤ 5				
Drug dependence in 2016–2018					
No evidence of dependence	42	29	1.00		
Distant dependence	10	17	0.50	0.23–1.07	.074
Recent dependence	16	27	0.90	0.46–1.78	.769

Abbreviations: DAA, direct‐acting antiviral; HCV, hepatitis C virus; IQR, interquartile range; LHD, local health district.

## 4 DISCUSSION

This study provides state‐level data on HCV care cascade among PLHIV in NSW, Australia, demonstrating the favourable impact of unrestricted access to DAA therapy on HCV testing and linkage to care. People living with HIV/HCV coinfection were highly engaged in the HCV testing continuum with the vast majority of the population receiving HCV RNA testing since 2010. HCV treatment uptake varied from suboptimal levels in pre‐DAA era to high levels post‐availability of DAAs, with a nearly 10‐fold increase in proportion of treatment uptake. These findings have important implications for HCV elimination efforts in Australia and elsewhere and highlight the success of the Australian public health approach to HCV: Broad testing and unrestricted DAA therapy access for all adults with chronic HCV infection.^23^, ^24^

The present study is one of the few population‐level studies that describes and evaluates both testing and treatment components of the HCV cascade of care among PLHIV. During eight years from 2010 to 2018, among those who ever had an HCV notification, 91% had HCV RNA testing and among those with an indicator of chronic HCV infection, 74% received HCV treatment, a transformation from the 7% treated in the pre‐DAA era. These findings are comparable with what was observed during six years in the British Columbia Hepatitis Testers Cohort (BC‐HTC) study among PLHIV: 90% HCV RNA testing among all HCV diagnosed individuals, and a 62% HCV treatment uptake among HCV RNA positive people.^14^

In the DAA era (2016–2018), both overall HCV treatment uptake and time from HCV diagnosis to treatment uptake substantially improved. A recent systematic review^24^ has reported variable levels of DAA treatment uptake among people living with HIV/HCV coinfection from 18% in the United States^25^, ^26^ and 21% in Switzerland,^27^ to 44% in the Netherlands,^28^ and 62% in British Columbia, Canada.^14^ Although DAA treatment uptake among people living with HIV/HCV coinfection was considered to be relatively high in three of these four studies, they are lower than what we have observed in Australia. Along with the great impact of rapid DAA scale‐up, the higher levels of treatment uptake in NSW compared with other settings can be explained by the fact that a high proportion of PLHIV (85%) are linked to and retained in care in Australia.^17^

This study further confirms that DAA treatment uptake in a population‐level linkage study is similar to those found in HIV/HCV coinfection clinic‐based cohort studies in Australia. CEASE (The Control and Elimination of HCV from HIV‐infected individuals within Australia)^11^ was an Australian study among HIV/HCV coinfected populations. Following universal access to DAA therapies, treatment uptake increased from 7% and 11% in 2014 and 2015, respectively, to 80% in 2016 in CEASE study.^11^ This resulted in a substantial decline in HCV viremic prevalence from 82% in 2014 to 8% in 2018 among this study population.^11^

We also identified the characteristics of people living with HIV/HCV coinfection in NSW who did not progress to testing or treatment, that could be used to optimize interventions to further improve the gaps in the continuum. Although HCV RNA testing has been high among PLHIV in the care cascade in NSW and has further improved in DAA era, there remain small but important gaps. Younger people were more likely to have HCV RNA testing meaning that older cohorts of PLHIV are left behind in the testing continuum, and therefore, less likely to be aware of their HCV infection status. We further observed that women and those who were HCV notified in rural regions are less likely to have HCV RNA testing. No factor was observed to be associated with not receiving DAA therapy among PLHIV in NSW; however, given the small number of untreated people in DAA era (*n* = 68), the sample size may have been under powered to draw any meaningful conclusion. The data was also limited by the relatively small number of demographic and behavioural characteristics collected, especially related to injecting drug use. Larger studies with more detailed information may be better able to tease out potential reasons for not receiving DAA therapy among PLHIV. Strategies aiming to reduce vulnerabilities among women living with HIV such as scaling up HCV testing in settings where women seek care for sexual and reproductive health can help health providers close the gap from HCV diagnosis to HCV RNA testing among women. Further, barriers to HCV screening and testing in rural regions need to be addressed. Strategies like point‐of‐care technologies along with continuous education for care providers can improve progression to HCV RNA testing.

This study has several limitations. First, our findings may not be nationally representative as the study has been carried out in one jurisdiction in Australia. However, NSW has been reported to have the highest proportion of people with chronic HCV (36%) in Australia by the end of 2017.^17^ Further, the proportion of people with chronic HCV infection initiating DAA therapy in 2017 in NSW (12%) has been reported to be similar to overall DAA uptake at the national level (12%) in Australia.^17^ Second, HCV treatment uptake was estimated among people with an indicator of chronic HCV infection (i.e. those with records of genotype testing or those who were dispensed HCV treatment). This may have potentially underestimated the denominator of chronically infected population as some people who were chronically infected might have never received genotype testing or treatment. However, this limitation is balanced against the high proportion of HCV RNA testing among our study population. Third, information on the proportion of individuals that completed HCV treatment as well as adherence and treatment outcomes were unavailable. This information provides insight into whether patients are transitioning through the stages of care or not, which would inform need for interventions to enhance treatment completion. Fourth, information on additional covariates such as HIV/HCV transmission patterns, fibrosis stage, etc. was not available. Including these factors could have allowed a better characterization of people living with HIV/HCV coinfection.

In conclusion, people living with HIV/HCV coinfection have progressed well through the testing continuum in NSW with nearly 10‐fold increase in HCV treatment uptake following the unrestricted availability of DAAs. Further efforts to reach those who have been lost to care after HCV diagnosis and those who are hardly reached are needed to close the remained gap in the treatment uptake among this population. In order to achieve the WHO HCV elimination targets, NSW needs to work on removing barriers to HCV testing, expanding treatment to a variety of settings and ensure that continuous education alongside harm reduction services is in place to prevent new HCV infections or reinfection after treatment among PLHIV.

## 5 CONFLICT OF INTEREST

GJD reports grants from Gilead Sciences, grants from Abbvie, grants from Merck, outside the submitted work. GVM reports grants from Gilead, grants from Abbvie, outside the submitted work. All other authors declare no competing interests.

## AUTHORS’ CONTRIBUTION

SHH, MA, GJD, MM and GVM contributed to the study conception and design. SHH, MA, HV, GJD and ST contributed to data analysis and interpretation of the findings. SHH drafted the original manuscript under supervision of MA and GJD. MM and GVM critically reviewed the manuscript. All authors contributed toward revision and approval of the final manuscript.

## Supporting information

Table S1‐S2

## Data Availability

This publication involved information collected by population‐based health administration registries. Data used for this research cannot be deposited on servers other than those approved by ethics committees. This publication has used highly sensitive health information through linkage of several administrative datasets. De‐identified linked information has been provided to the research team under strict privacy regulations. Except in the form of conclusions drawn from the data, researchers do not have permission to disclose any data to any person other than those authorized for the research project.
