# Supplementary material for: Evaluation of the hepatitis C cascade of care among people living with HIV in New South Wales, Australia: A data linkage study
Source: J Viral Hepat. 2022 Feb 25;29(4):271–9. doi: 10.1111/jvh.13658 (PMC9306975; doi:10.1111/jvh.13658)
Supplement: Supplementary file 1 — Table S1‐S2 [file JVH-29-271-s001.docx]

**Supplementary Table 1.** ICD-10 definitions used to infer the presence of alcohol-use disorder among people living with HIV/HCV coinfection in NSW, Australia

| **ICD-10** | **Description** |
| --- | --- |
| E24.4 | Alcohol induced Pseudo-Cushing’s syndrome |
| F10 | Mental and behavioural disorders due to use of alcohol |
| G31.2 | Degeneration of nervous system due to alcohol |
| G62.1 | Alcoholic polyneuropathy |
| I42.6 | Alcoholic cardiomyopathy |
| G72.1 | Alcoholic myopathy |
| Z50.2 | Alcohol rehabilitation |
| Z71.4 | Alcohol abuse counselling and surveillance |

**Supplementary Table 2.** ICD-10 definitions used to identify injecting drug use-related hospital presentations among people living with HIV/HCV coinfection in NSW, Australia

| **ICD-10** | **Description** |
| --- | --- |
| A40 | Streptococcal sepsis |
| A41 | Other sepsis |
| A48.0 | Other bacterial diseases, not elsewhere classified (gas gangrene) |
| B37.6 | Candidiasis, candida endocarditis |
| F11 | Mental and behavioural disorders due to use of opioids |
| F13 | Mental and behavioural disorders due to sedatives or hypnotics |
| F14 | Mental and behavioural disorders due to use of cocaine |
| F15 | Mental and behavioural disorders due to use of other stimulants, including caffeine |
| F19 | Mental and behavioural disorders due to multiple drug use and use of other psychoactive substances |
| G06 | Intracranial and intraspinal abscess and granuloma |
| G09 | Sequelae of inflammatory disease of central nervous system |
| I26.9 | Pulmonary embolism, pulmonary embolism without mention of acute or pulmonale |
| I33 | Acute and subacute endocarditis |
| I34 | Nonrheumatic mitral valve disorders |
| I35 | Nonrheumatic aortic valve disorders |
| I36 | Nonrheumatic tricuspid valve disorders |
| I37 | Pulmonary valve disorders |
| I38 | Endocarditis, valve unspecified |
| I39 | Endocarditis and heart valve disorders in diseases classified elsewhere |
| I40.0 | Acute myocarditis, infective myocarditis |
| I80 | Phlebitis and thrombophlebitis |
| K63.0 | Other diseases of the intestine, abscess of intestine |
| K65.0 | Peritonitis, acute peritonitis |
| K75.0 | Other inflammatory liver disease, abscess of liver |
| L02 | Cutaneous abscess, furuncle and carbuncle |
| L03 | Cellulitis |
| L97 | Ulcer of lower limb, not elsewhere classified |
| L98.8 | Other disorders of skin and subcutaneous tissue, not elsewhere classified, other specified disorders of skin and subcutaneous tissue |
| M54.0 | Dorsalgia, panniculitis affecting regions of neck and back |
| M72.6 | Fibroblastic disorders, necrotizing fasciitis |
| M79.3 | Other soft tissue disorders, not elsewhere classified (panniculitis, unspecified) |
| M86 | Osteomyelitis |
| M89.9 | Other disorders of bone, disorder of bone, unspecified |
| N10 | Acute tubulo-interstitial nephritis |
| R02 | Gangrene, not elsewhere classified |
| R57.2 | Shock, not elsewhere classified, septic shock |
| R65.1 | Systemic Inflammatory Response Syndrome of infectious origin with organ failure |
| R65.9 | Systemic Inflammatory Response Syndrome, unspecified |
| R78.1 | Finding of opiate drug in blood |
| R78.2 | Finding of cocaine in blood |
| T38.7 | Androgens and anabolic congeners |
| T40.0 | Poisoning by narcotics and psychodysleptics, opium |
| T40.1 | Poisoning by narcotics and psychodysleptics, heroin |
| T40.2 | Poisoning by narcotics and psychodysleptics, other opioids (codeine/morphine) |
| T40.3 | Poisoning by narcotics and psychodysleptics, methadone |
| T40.4 | Poisoning by narcotics and psychodysleptics, other synthetic narcotics (pethidine) |
| T40.5 | Poisoning by narcotics and psychodysleptics, cocaine |
| T40.6 | Poisoning by narcotics and psychodysleptics, other and unspecified narcotics |
| T40.8 | Poisoning by narcotics and psychodysleptics, lysergide (LSD) |
| T41.2 | Poisoning by anaesthetics and therapeutic gases, other and unspecified general anaesthetics |
| T42.3 | Poisoning by antiepileptic, sedative-hypnotic and antiparkinsonism drugs, barbiturates |
| T42.4 | Poisoning by antiepileptic, sedative-hypnotic and antiparkinsonism drugs, benzodiazepines |
| T42.5 | Poisoning by antiepileptic, sedative-hypnotic and antiparkinsonism drugs, mixed antiepileptics, not elsewhere classified |
| T42.6 | Poisoning by antiepileptic, sedative-hypnotic and antiparkinsonism drugs, other antiepileptic and sedative-hypnotic drugs |
| T42.7 | Poisoning by antiepileptic, sedative-hypnotic and antiparkinsonism drugs, antiepileptic and sedative-hypnotic drugs, unspecified |
| T42.8 | Poisoning by antiepileptic, sedative-hypnotic and antiparkinsonism drugs, antiparkinsonism drugs and other central muscle-tone depressants |
| T43.6 | Poisoning by psychotropic drugs, not elsewhere classified, psychostimulants with abuse potential |
| T43.8 | Poisoning by psychotropic drugs, not elsewhere classified, other psychotropic drugs, not elsewhere classified |
| T43.9 | Poisoning by psychotropic drugs, not elsewhere classified, psychotropic drug, unspecified |
| T50.7 | Poisoning by psychotropic drugs, not elsewhere classified, analeptics and opioid receptor antagonists |
| X41 | Accidental poisoning by and exposure to antiepileptic, sedative-hypnotic, antiparkinsonism and psychotropic drugs, not elsewhere classified |
| X61 | Intentional self-poisoning by and exposure to antiepileptic, sedative-hypnotic, antiparkinsonism and psychotropic drugs, not elsewhere classified |
| Y11 | Poisoning by and exposure to antiepileptic, sedative-hypnotic, antiparkinsonism and psychotropic drugs, not elsewhere classified, undetermined intent |
